# A Morphological Evaluation of the Antibiofilm Activity on an Implant Surface Using a New Electric Device: An In Vitro Study

**DOI:** 10.3390/dj13040140

**Published:** 2025-03-25

**Authors:** Gianluca Botticelli, Giovanni Falisi, Sofia Rastelli, Enzo Iacomino, Angelo Bruni, Davide Gerardi, Giuseppe Di Fabio, Marco Severino, Sara Bernardi

**Affiliations:** 1 Department of Life, Health and Environmental Sciences, University of L’Aquila, 67100 L’Aquila, Italy; gianluca.botticelli@univaq.it (G.B.); giovanni.falisi@univaq.it (G.F.); sofia.rastelli@graduate.univaq.it (S.R.); enzo.iacomino@graduate.univaq.it (E.I.); giuseppe.difabio@hotmail.com (G.D.F.); 2 Department of Biotechnological and Applied Clinical Sciences, University of L’Aquila, 67100 L’Aquila, Italy; angelo.bruni@graduate.univaq.it; 3 Department of Medicine, School of Medicine, Odontostomatological University Centre, University of Perugia, S. Andrea delle Fratte, 06132 Perugia, Italy; marco.severino@unipg.it

**Keywords:** dental implants, peri-implantitis, oral biofilm, decontamination systems, electrostatic decontamination

## Abstract

**Background:** Peri-implantitis, the most prevalent cause of implant failure, is a multifaceted issue that is influenced by various factors that promote biofilm formation around the implant. Although various innovative methods for microbiological decontamination of dental implants exist, a universally accepted standard protocol has not yet been established. However, the potential of a device that generates an electric current (Ximplant^®^) in reducing the survival of microorganisms within the biofilm is a promising development. **Methods:** In this in vitro study, five dental implants, contaminated using a microbial culture from a sample of saliva of a patient suffering from peri-implantitis, were decontaminated using the Ximplant^®^ peri-implantitis protocol. The experimental conditions included a simulated peri-implant site and a subsequent fluorescent assessment of the Live/Dead microbial population. **Results:** The qualitative and quantitative image analyses showed a predominant dead light signal on the treated sample, demonstrating the potential efficacy of applying the electrostatic field to the contaminated implant surface in reducing the viability of the microorganisms within the biofilm around dental implants. **Conclusions:** These findings could inspire a new era in peri-implantitis treatment.

## 1. Introduction

Dental implants, a widely used solution for replacing missing teeth, have shown a high success rate in recent research, making them a predictable and suitable treatment for most patients [1].

However, the long-term efficacy of implant therapy depends on maintaining proper oral hygiene practices, particularly the correct application of home and professional hygiene standards for peri-implant tissues [2]. The risk of developing pathological conditions in the peri-implant tissues is often associated with poor oral hygiene or prosthetic-related issues, which trigger inflammatory responses that are exacerbated by genetic polymorphisms of different cytokines, such as the interleukine family (IL-1), in both soft and hard tissues [3]. 

Various protocols for peri-implant surface decontamination exist to limit disease progression and restore proper hygiene around implants, ensuring their function and longevity [4]. Several methods of morphological characterization can be employed to study the microbiological properties of biofilms. The morphological approach helps identify the microorganisms that are present and the chemical and physical factors that may influence the biofilm’s adhesion to different surfaces in the oral cavity [5]. These surfaces include natural teeth and dental implant surfaces, where roughness can impact both the success of osteointegration and the adhesion of oral biofilms [6]. The primary decontamination methods include non-surgical treatments using ultrasonic scalers and curettes, which may require more invasive interventions, such as surgical flap procedures for exploration and decontamination [7], and the use of home care maintenance medication such as mouthwashes and gels [8]. Non-surgical therapy often precedes surgical peri-implant treatments, including ablative or regenerative procedures for soft and hard tissues [9].

The future of peri-implant disease treatment looks promising, with the application of several innovative decontamination techniques during the initial non-surgical phase. These include laser therapy [10], photodynamic therapy, and the application of alternating currents. Each of these techniques offers a new avenue for effective treatment, instilling optimism in the dental community. 

Erbium lasers (Er) are the most studied for treating peri-implant diseases. They utilize a wavelength that allows for the vaporization of water in biological tissues and bacterial biofilm [11]. They are particularly effective in decontaminating implant surfaces and removing necrotic or inflamed tissue, minimizing the damage to the soft tissues thanks to the absence of thermal damage to titanium implant surfaces, promoting rapid tissue healing, and effectively removing bacterial biofilm and calculus [12].

Diode lasers are mainly used for their bactericidal properties and their ability to reduce inflammation in the soft tissues surrounding implants, in combination with other therapies to improve treatment outcomes: they are effective in reducing bleeding during treatment, decontaminating soft tissues, reducing inflammation, and promoting the healing of peri-implant soft tissues [13].

Neodymium–YAG (Nd:YAG) lasers penetrate deeper into soft tissues than other lasers, making them useful for deep decontamination of peri-implant pockets and reducing inflammation [14].

Antimicrobial photodynamic therapy (aPDT) is an antimicrobial alternative method of oral biofilm decontamination. An example of photodynamic therapy involves combining visible light (VIS) and water-filtered infrared-A with a photosensitizer (PS). This therapy uses a light source to excite the PS to a high-energy triplet state in the presence of molecular oxygen, generating reactive oxygen species (ROS). These ROS affect the metabolic activity of bacterial cells, leading to the disruption of biofilm [15].

Finally, the interest in using the electrical field is increasing due to the potential ability that this method has to disrupt oral biofilm [16]: an alternating current can produce electrostatic forces that disturb the adhesion of the biofilm to the surfaces of the tooth or dental implant, leading to a disruption of the extracellular matrix and, consequently, to the alteration of the oral biofilm and the viability of microorganisms [17]. The low direct current efficiently reduces the biofilm’s viability, causing a direct disruption on the titanium implant surface [18].

This in vitro study aims to assess the effects of alternating current on removing the typical oral biofilm causing peri-implantitis. The potential application of this innovative method in clinical practice is an intriguing prospect that could significantly impact the future of dental care.

## 2. Materials and Methods

### 2.1. Study Design

This study was an in vitro study, which included different operative phases: (1) saliva sampling from a patient suffering from peri-implantitis; (2) the cultivation of the related microorganisms; (3) the contamination of one implant surface and the related assessment of microbial growth; (4) the contamination of the implant surfaces in a simulated peri-implant site; (5) the application of the Ximplant protocol; and (6) the fluorescent assessment of the microbial population.

### 2.2. Sample Collection

A sample of saliva and plaque above and below the gingival line was collected from a patient with established periodontal disease. On the morning of the collection, the patient was advised to fast and not to perform his usual home oral hygiene routine.

Saliva was collected by placing the sample in a sterile test tube for 5 min. Mucobacterial plaque was collected from the supragingival and subgingival spaces using a sterile curette. The collected samples were stored at −20 °C. Subsequently, they were thawed at room temperature, enriched using a BHI (Brain Heart Infusion—Liofilchem, Roseto degli Abruzzi (TE), Italy) culture medium for 24 h, and incubated at 37 °C.

The initial seeding of the enriched samples on Agar culture media (Liofilchem) was followed by incubation at 37 °C in an aerobic atmosphere, ensuring optimal conditions for growth. The identification of the microbial species in the post-incubation media culture was performed using the matrix-assisted laser desorption/ionization mass spectrometry technique (BRUKER IVD Maldi Biotyper System), known by the acronym MALDI, a highly accurate and reliable method.

### 2.3. Implant Fixture Contamination: Assessment of the Microbial Load and Realization of the Peri-Implantitis Site Replica

A scanning electron microscope (SEM) was used to assess the effective microbial growth on the implant surface.

The in vitro procedure for the contamination of implant fixtures (Global D—TwinKon^®^, Brignais, France) was performed in vitro in a sterile field and with sterile instruments, using disposable surgical gloves, surgical forceps, and an implant transporter. A first sterile fixture was cultivated in a BHI enrichment broth containing the sampled plaque for 24 h at 37 °C. Afterward, it was processed for scanning electron microscope observation to assess the microbial growth. Briefly, the contaminated implant was fixed in glutaraldehyde 2% and, after 48 h, submitted to dehydration in an ascending series of alcohols (50%, 75%, 95%, 100%). After 48 h of drying on absorbent paper, the sample was observed using an SEM (GEMINI_SEM, Zeiss, Germany) [16,19,20]. The surface was randomly observed in different locations at different degrees of magnification in secondary electron (SE) mode. The used parameters were an acceleration voltage (AV) of 5.00 kV and working distance between 8.4 and 3.7 mm.

The implant fixtures (n = 5) were inserted into a BHI enrichment broth containing plaque and saliva collected from the periodontal patient for 120 h at 37 °C in an uncontrolled atmosphere. Every 24 h, half the volume of the enrichment medium was replaced with a new BHI medium to provide it with new nutrients.

**Figure 1:**
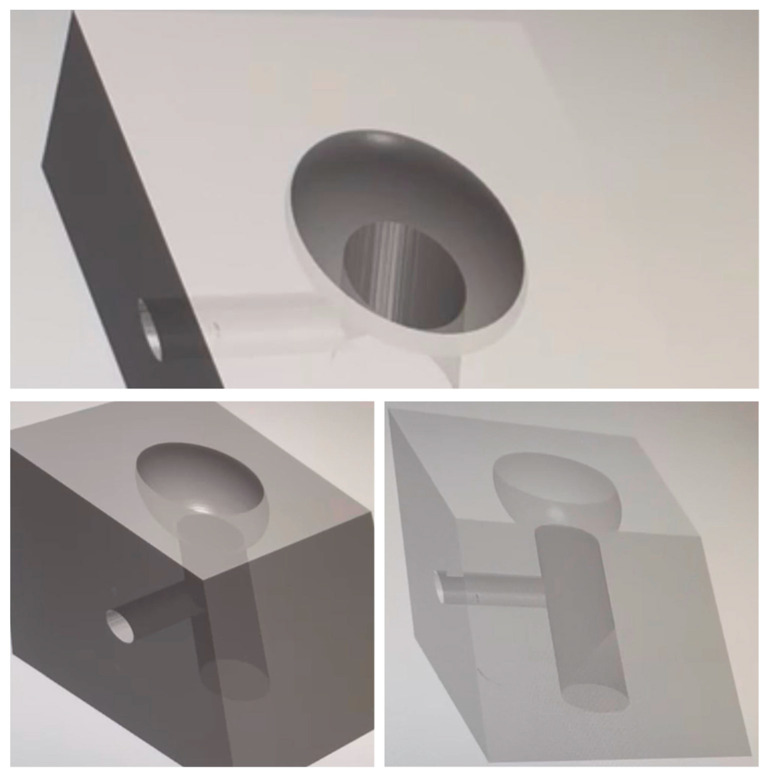
CAD procedure for the bone defect resin blocks.

Our study utilized the CAD/CAM procedure to create a solid structure that accurately replicated the implant site affected by peri-implantitis. This structure, which exhibited an open “bowl-shaped” bone defect, as per the classification of L. Vanden Bogaerde (2004), was designed to serve as a model for our in vitro experiments [21]. The three-dimensional bases were crafted from transparent solid resin using the Exocad^®^ design software (Netfabb, vers 7.4.0, Exocad GmbH, Darmstadt, Germany), following the mathematics of the Twincon^®^—Global D fixture for the implant screwing area. The printing was performed with a FormLab^®^ 3D printer (Sinterit, Krakow, Poland). A channel was incorporated on the lateral surface of the 3D base (Figure 1) to facilitate the connection with the Ximplant machine (LED S.P.A., Latina, Italy).

Prior to use, each base underwent a rigorous sterilization process using sterile instruments and the ID-212 (Durr Dental) cold technique, ensuring a pristine environment. The bases were then left to dry at room temperature in a sterile environment. Finally, the contaminated fixture was removed from the culture broth using sterile instruments and securely screwed into the site, created previously in the 3D resin base.

### 2.4. Ximplant and Peri-Implantitis Protocol

The “peri-implantitis” protocol was chosen to remove the bacterial biofilm from the implant surface. This was achieved by screwing the contaminated fixture into the 3D base using the appropriate implant carrier, exposing approximately half of its length. The alternating electric current supplied by the Ximplant machine (LED S.P.A., Latina, Italy) was then used to decontaminate the surface of the fixture that was affected by the peri-implant bacterial inflammatory pathology.

The Ximplant machine (LED S.P.A., Latina, Italy) consists of a unipolar electrode and a “bunch” electrode, which is an electroconductive stick that is placed in the patient’s hand in clinical practices to close the circuit. This closure allows pulses of electric current to pass through, a process that is central to the decontamination protocol of our study.

**Figure 2:**
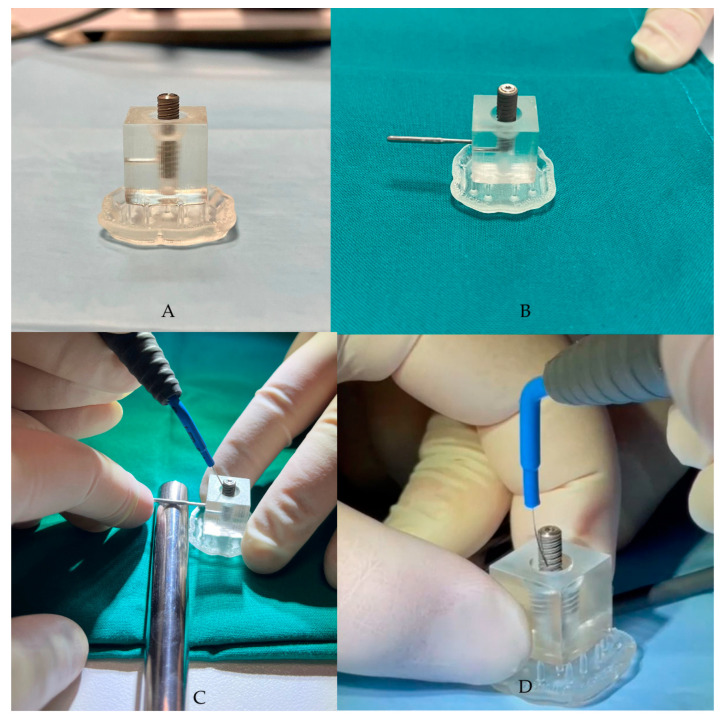
(**A**,**B**) The contaminated fixture placed in the resin block, created using CAD/CAM technology. (**C**,**D**) Ximplant applications.

The third phase involves the application of alternating electric current, supplied by the Ximplant machine (Figure 2) through the dedicated “peri-implantitis program,” which includes four active cycles of 3 s alternating with 2 s of pause (alternating electric current at 625 kHz, 260 Vpp, 15 W, manufacturer’s declared data). The alternating electric current is delivered to the fixture using the linear tip produced by the same company. In this in vitro study, the “peri-implantitis” protocol was performed 5 times on the fixture.

The Ximplant peri-implantitis protocol includes four cycles lasting 3 s each, which are interspersed with a 2 s pause for each treatment. The alternating current (AC) flows through the unipolar 92 electrode with a sine wave at 625 kHz, 260 Vpp peak-to-peak voltage, and 15 W power (manufacturer’s declared data).

Once the decontamination protocol was completed, the fixture was unscrewed using a sterile implant carrier and inserted into a sterile Eppendorf tube. It was then colored and observed under a fluorescence microscope.

The indications in the documentation submitted for the obtained CE mark describe XImplant as a medical device intended for temporary use in the prevention, reduction, and inactivation of the bacterial load in patients with implant prostheses that are affected by mucositis and/or peri-implantitis. The use of the device “in vivo” is indicated in patients over 18 years of age and is contraindicated in pregnant subjects, subjects with pacemakers, subjects with severe blood pressure imbalances, subjects suffering from epilepsy, and subjects with sensitivity to thermal pain. The values of the alternating electric current, as reported by the manufacturer, are a maximum power 20 W and frequency 625 kHz, which do not represent a danger for the patient.

### 2.5. Live/Dead Staining, Microscopy Observation, and Quantitative Image Analysis

After treatment using the Ximplant machine, in accordance with the manufacturer’s instructions for the staining kit, the implant fixtures were collected with sterile instruments and subjected to the Live/Dead staining protocol “fresh” without surface treatment, as indicated by the manufacturer.

The LIVE/DEAD^®^ BacLight Bacterial Viability L7012 kit (Thermo Fischer Scientific, New York, NY, USA) was used. This bacterial viability kit for microscopy and quantitative analysis includes two separate solutions of SYTO 9 dyes (3.34 mM) and propidium iodide (20 mM) in 300 μL of DMSO. The separate staining components allow for flexibility in the optimization and facilitate the calibration of bacterial fluorescence for quantitative procedures. Following treatment of the contaminated implant surface with the alternating electric current delivered by the Ximplant, bacterial cells with a compromised membrane (dead or severely compromised) are stained red by propidium iodide. In contrast, cells with an intact membrane (viable) are stained green by SYTO™ 9.

Afterward, the stained samples were observed using a Zeiss Axio Imager M2 microscope, and the images were captured using AxioCam 503 mono. Moreover, AxioCam 305 color cameras use fluorescence filters without shift. The used magnifications were 2.5× and 10×.

The captured images were submitted to quantitative image analysis using Fiji open source software (Vers. 2.16.0) [22]. The quantitative image analysis determined the number of detectable particles that were identified as bacteria. The protocol used for segmentation and particle analysis followed the “Live/Dead Quantification Using Fiji” guide [23].

Specifically, the protocol of quantitative image analysis includes the following steps:

1. Selection of the captured picture;
2. Splitting of the colors into the red and green channels to quantify the signal;
3. Obtainment of a single channel 8-bit type image, which is a gray-scale type of image;
4. Cleaning of noise from the image;
5. Segmentation of the image, transforming it into a binary black-and-white image;
6. Counting of the segmented cells.

In our images, the considered area was measured first to ensure the uniformity of the considered samples and then processed for the quantitative image analysis.

The collected counts of live and dead microbial cells were then processed further for statistical analysis.

### 2.6. Statistical Analysis

Our null hypothesis was that there would be no difference between the dead bacterial particles and the live bacterial particle numbers. After assessing the normal distribution of the data, the Wilcoxon matched-pairs signed rank test was used to assess the difference between the two considered groups. If the *p*-value resulted in ≤0.05, the null hypothesis was rejected. GraphPad Prism version 10.4.0 (621) for Windows (GraphPad Software, Boston, MA, USA) was used for the analysis.

### 2.7. Ethics

Ethical approval is not required due to the mainly in vitro nature of the experiment, and the noninvasive saliva sampling procedure. This study is also part of a project on the use of the electric machine for implant decontamination, approved by the IRB of Albanian University, approval code: n prot. 671, date of approval: 21 November 2022.

## 3. Results

### 3.1. Initial Microbial Population Assessment

The MALDI analysis assessed the presence of the following microorganisms in the saliva-cultured sample: *Streptococcus parasanguinis*, *Lactobacillus lactis*, *Streptococcus oralis*, *Neisseria subflava*, *Candida albicans*, and *Streptococcus salivarius*.

### 3.2. SEM Assessment of Microbial Growth

**Figure 3:**
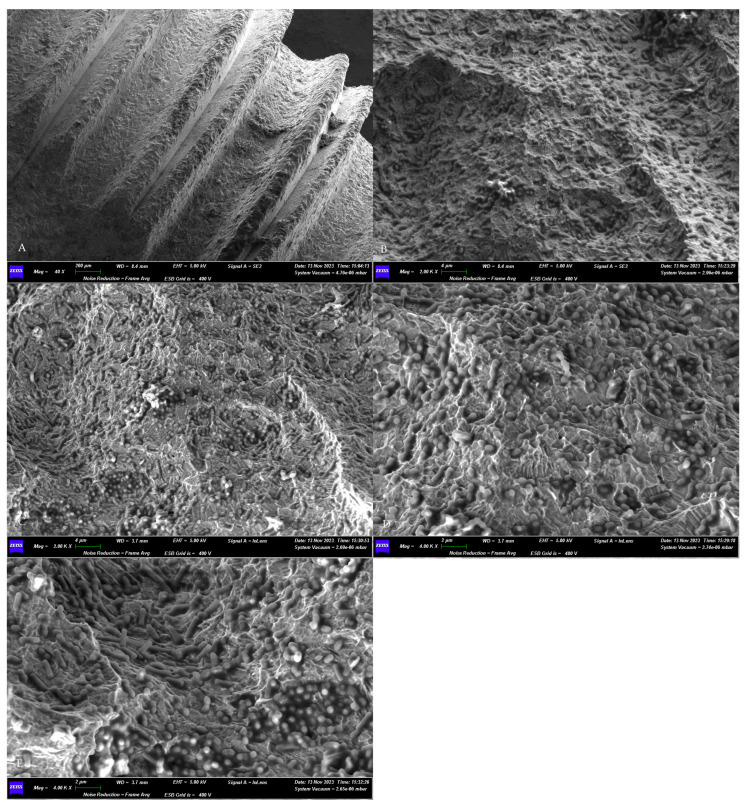
SEM assessment of the microbial growth on contaminated implants surfaces. (**A**) Panoramic view of the sample. Magnification 40×. (**B**–**E**) Higher magnifications (4000× and 2000×) of the biofilm matrix containing numerous microbial communities of different shapes. GEMINI_SEM, Zeiss, Germany. In our previous study [16], the treated surfaces, observed using SEM, appeared without a microbial matrix and microorganisms.

The morphological assessment of the microbial growth on the titanium surface of the sterile implant obtained a positive result. The SEM observations revealed the presence of a dense microbial matrix, enveloping numerous microorganisms of several morphologies (Figure 3).

### 3.3. Qualitative Fluorescence Observation

**Figure 4:**
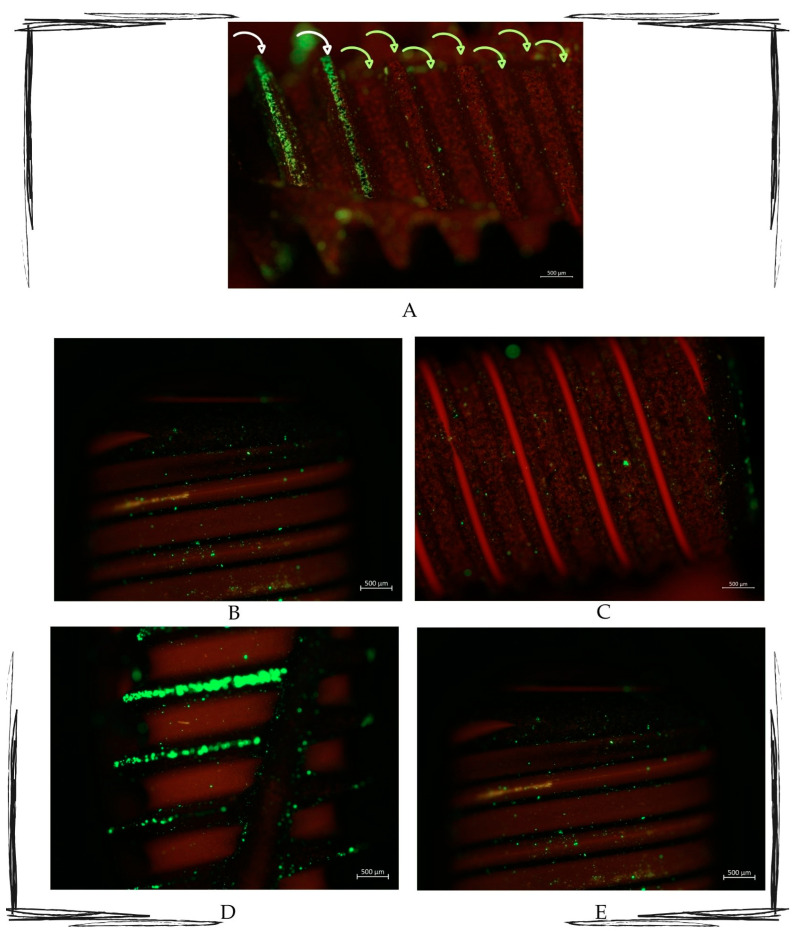
Multipaneled fluorescent microcopy picture showing the most representative observed samples. (**A**) Image showing how the untreated area (indicated by the white arrows) is full of green light particles, whilst the treated part (indicated by the green arrows) presents the red signal as the most representative. (**B**–**E**) Fluorescence-treated samples. All pictures show treated areas, which present both red and green particles, with a marked red signal in all the samples. Zeiss Axio Imager M2. Magnification 10×.

The qualitative fluorescence observations revealed that the surfaces that were submitted to the Ximplant protocol emitted a red signal, while those that were not treated emitted a green signal (Figure 4).

### 3.4. Quantitative Image Analysis and Statistical Results

A total of 14 images of five contaminated implants were considered for the quantitative image analysis. The mean area of the considered fluorescent images, measured using Image J (8Vers. 1.54 k), was 0.15 mm^2^. The mean number of green-light-emitting signal particles was 1533.5. The mean presence of the red-light-emitting signal particles was 4394 (Table 1).

**Figure 5:**
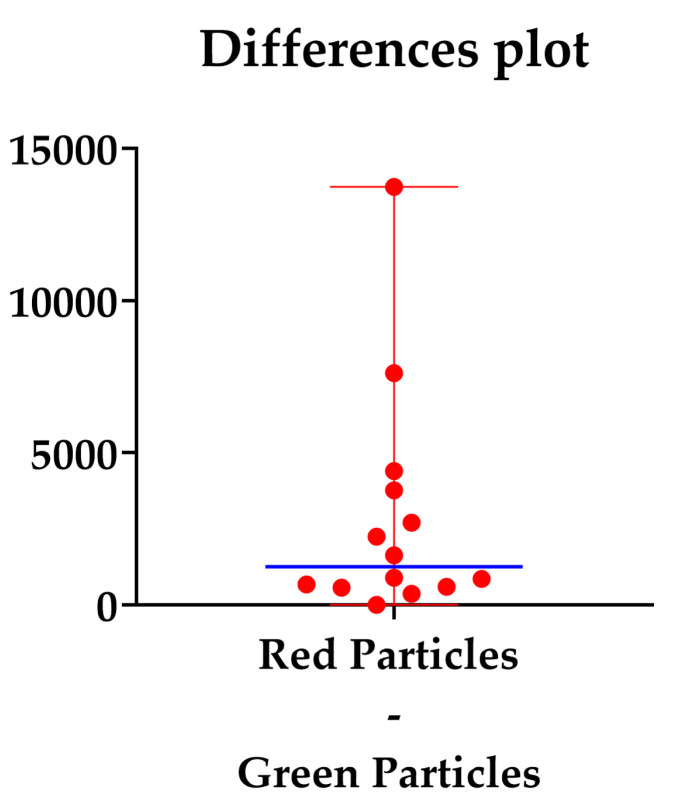
Difference plot between the red and green counted particles. The blue line represents the median differences.

The median difference was 1261, as shown in Figure 5.

The *p*-value of the Wilcoxon matched-pairs signed rank test was <0.05 (Table 2), so the null hypothesis was rejected, and the differences between the live and dead signals (measured as the viable and not viable microorganisms after the XIMPLANT procedure) were statistically significant.

## 4. Discussion

### 4.1. Peri-Implantitis: The New Emerging Dental Disease That Should Be Avoided as Much as Possible

Implant therapies have represented a solution for years, also in very complex clinical situations, to rehabilitate the edentulous arches. Indeed, titanium fixtures improved patients’ quality of life, from a single missing tooth to a complete edentulous crest [24]. However, this therapy is not immune to the action of the microbial community that is hosted in the oral cavity. For many years, periodontologists, dental hygienists, and implantologists have been observing the occurrence of an inflammatory disease affecting the tissues surrounding the implant: peri-implantitis [25].

This condition mainly involves microbial etiopathogeneses, but on some occasions, other factors can generate the inflammatory process, such as metal debris. Indeed, recent studies [26,27] proposed the hypothesis that the residual metal debris could be derived from the drill’s tip drill from the sleeves of the guide templates that are used in guided implantology, and metal residuals in the bone site were found following procedures with poor irrigation and consumption of the tips due to continuous usage. Metal debris triggers inflammatory processes around endo-osseous metal prostheses, including the titanium dental fixture [27], and generate peri-implantitis.

Peri-implantitis is still not considered to be among the Global Burden of Diseases but affects one out of five individuals who are rehabilitated using implant therapy [28,29,30,31]. Its progression is less linear than periodontitis, and the available treatments are not fully predictable. These treatments increase dental care costs, and if not effective, the loss of an implant can occur, thus impairing the quality of life of the patient [32]. 

The physiology of a peri-implant biofilm includes a very delicate balance between the microbial communities, showing the existence of an ecological niche, which includes a network of different species of microorganisms living with a certain degree of stability. In case of disease occurrence, this balance inevitably changes. Indeed, Zhaugh et al. showed that in unhealthy individuals, the network and stability of supra-gingival microorganisms are stronger than those of sub-gingival microbial communities [33]. The shift and the aberrant changes in the microbial network influence the progression of peri-implantitis.

These data are confirmed by the study by Banu Raza et al., which summarized the microbial community species around healthy, mucositis, peri-implantitis, and failing implant sites [34].

The predominant microorganisms in healthy peri-implant sites were *Actinomyces*, *Capnocytophaga*, *Neisseria*, *Rothia*, and *Streptococcus.*

In peri-implant sites affected by mucositis, the predominant species was *Eikenella Corrodens*, with the co-current presence of *Porphyromonas gingivalis*, *Tannerella forsythia*, and *Prevotella intermedia.*

This microbial network is, therefore, the base of the early onset of peri-implantitis. In peri-implantitis sites, the peri-implant-related complex (*Prevotella intermedia*, *Prevotella nigrescens*, *Porphyromonas gingivalis*, *Fusobacterium nucleatum*, *Treponella Denticola*, and *Tannerella forsythia*), microbes, *Eubacteria* (sp.), *Filifactor alocis*, and *Parvimonas Micra* are also found.

Regarding failing implants, those that failed early presented a smaller variety of microbial populations *(Fusobacterium*, *Aggregatibacter*, *Gemella)* than those that failed late *(Tannerella*, *Treponema*, *Fusobacterium*, *Porphyromonas*, *Fretibacterium*, and *Desulfobulbus).*

All these different microorganisms regulate their biofilm formation through so-called “Quorum sensing”, an intercellular molecule-mediated signal [35].

These molecules are responsible for inter-species interaction and surface adhesion [35].

The species found in the donated sample belonged to the predominant species that are usually found in healthy peri-implant sites. However, the sample derived from saliva, probably showing the ecological niche in the oral mucosa and saliva, differed from the one around the implants. Therefore, attention also should be given to the specific site and how the substrate can influence the microbial network balance and its shifts.

The burden of peri-implantitis is significant from the biological, social, and economic perspectives. It necessitates specific actions from healthcare providers, including the implementation of preventive measures, early diagnosis, and appropriate therapy.

### 4.2. The Use of Electric and Electrolytic Effects to Remove Peri-Implant Biofilms as a Reliable Clinical Option

Dental implant therapy includes a biological cost; proper hygienic maintenance is mandatory to prevent fixture loss.

Over the years, disinfectants, local antimicrobial administration, glycine powder prophylaxis, and plastic tips on sonic and ultrasonic handpieces have been developed. They are currently used to temporarily remove the biofilm forming on implant-exposed surfaces [16,21].

All these therapeutic strategies are a legacy of periodontal therapy, which has been totally transposed by peri-implant therapy. They are effective in removing bacterial biofilm from the root surface but have poor results in removing it from the fixture’s surface.

In fact, the design of both the implant’s macro–microstructure and the morphology of the bone defect determines the difficulty of managing the infection due to the lack of complete eradication of the biofilm and toxic compounds, which results in poor osteointegration [36].

In a recent review by Giok et al. [37], the combined use of non-surgical interventions such as mechanical debris or photodynamic therapy with local or systemic antibiotics was reported to be effective. However, the use of local or systemic antibiotics has side effects such as antibiotic resistance development and further shift and changes in the ecological niche [37].

Instead, the use of physical methods for biofilm eradication is widely considered due to microorganisms’ capacity to develop resistance to antibiotics and disinfectants, as well as to other abiotic surfaces that are used in patients who are affected by cystic fibrosis [38].

The electric effects associated with biofilm reduction are the generation of electric forces acting on the biofilm adhesion forces, the generation of electrostatic forces, and the generation of oxygen and hydrogen [39].

Schlee et al. concluded that treating the implant surface with the electrolytic approach significantly increases the amount of radiographic bone and improves clinical parameters during 18 months of follow-up [40]. An in vitro study by Ratka et al. experimented with using electrolysis to decontaminate the implant surfaces, comparing it with air-polishing, resulting in more favorable results following the tested methodology [41]. An in vivo study by Bosshardt et al. confirmed the efficacy of electrolysis and subsequent regenerative procedures [42].

The use of electric and electrolytic fields can also be combined with administering antimicrobials. Studies have reported the possibility of increasing the permeability of bacterial membranes when the electric impulse is applied, facilitating the action of antimicrobials [43]. 

Our previous in vitro study showed, using SEM, the biofilm removal efficacy of Ximplant on a contaminated implant surface [16]. In our previous study, the absence of the biofilm matrix was appreciable in the SEM observations. However, the study’s limitations, such as the absence of a quantitative analysis and the condition of applying the Ximplant tip, led us to continue the project with a second step. 

The data presented in the current study represent the results of the second step of the project, which aimed to further assess the device’s microbicidal activity using fluorescent microscopy, differentiating living from dead microorganisms.

The quantitative image analysis revealed a significant difference between the living and dead microbial communities in the areas where the machine was applied. The resulting data, within the limitations of the quantitative image analysis, showed a significant efficacy of the application of Ximplant for biofilm removal, which is in agreement with our previous study [16] and with the available research in this field.

The experimental conditions also changed to simulate the typical morphology of a peri-implantitis site. In addition, the increased sample size allowed us to perform a quantitative image analysis.

The current study brings to “the table” the evidence, using Live/Dead fluorescence dye and quantitative image analysis, of the microbicidal efficacy of Ximplant on contaminated titanium surfaces, simulating the morphology of the peri-implantitis site.

### 4.3. Strengths and Limitation

The results of the detection and quantification of microorganisms in the oral biofilm are strongly influenced by the characteristics of the oral biofilm and the surface on which it adheres. Firstly, over 50% of the oral bacterial flora is not culturable [17], and the preparation of culturing techniques to identify bacteria in the biofilm on a specific substrate often requires laboratory procedures that affect the visualization of the sample [44]. The current evidence in the literature demonstrates that the characterization of oral biofilms and morphological methods are varied. Thus, researchers should choose the most suitable strategy depending on the investigation type, the oral biofilm’s different metabolic characteristics, and the microscopy techniques [5]. For this reason, in this study, the direct visualization and quantification of bacteria in their physiologically adherent state on the titanium surface of dental implants were preferred [45]. The detection mechanism of SYTO 9 green stain is based on the penetration of intact membranes and the interaction with the DNA- or RNA-contrasting chromatin [46]. The combination of SYTO 9 with PI allows for the assessment of bacterial viability through live cell fluorescence microscopy [47,48]. The limits of this study include the sample size and the limitation of the reliability of quantitative image analysis. Indeed, the threshold stage might depend on the operator. In addition, culture-dependent and -independent analyses would be helpful to support the quantitative image analysis results and are strongly encouraged.

In addition, the design of the study did not include a quantitative image analysis of untreated areas and a consequent statistical analysis, limiting the validity of the provided data.

Further in vitro and vivo studies, also using a negative control sample, animal models, and RCTs, are necessary to support these results.

## 5. Conclusions

Peri-implantitis is a relatively newly emerging disease involving at least one out of five individuals with dental implants. Using local and systemic antimicrobials is not fully effective and predictable and increases the risk of resistance development. For this reason, prevention strategies, early diagnosis, and the combined use of physical and chemical tools to remove a dysbiotic biofilm represent the available treatment options to avoid implant failure due to oral microbes and to manage the onset of peri-implantitis. Using innovative methods such as electrostatic fields could remove a significant amount of oral biofilm and restore the health condition of hard and soft tissues around dental implants.

## Figures and Tables

**Table 1:** Raw obtained data from the quantitative image analysis, including mean and standard deviation.

Imaging Sample	Area Squadre cm	Green Particles	Red Particles
A1	0.14	992	993
A2	0.13	960	8574
A3	0.16	4741	18,470
A4	0.18	4546	6176
B1	0.14	132	726
B2	0.14	972	1342
B3	0.13	946	1837
C1	0.15	841	1693
C2	0.16	1320	1893
C3	0.14	58	731
C4	0.16	1643	6043
D1	0.16	2614	4862
D2	0.16	948	3650
D3	0.15	756	4526
MEAN	0.15	1533.5	4394
STANDARD DEVIATION	0.01	1452.8	4724.2

**Table 2:** Median differences and Wilcoxon matched-pairs signed rank test.

Wilcoxon Matched-Pairs Signed Rank Test	
***p*** **value**	0.0001
**Exact or approximate *p* value?**	Exact
***p*** **value summary**	***
**Significantly different (*p* < 0.05)?**	Yes
**One- or two-tailed *p* value?**	Two-tailed
**Sum of positive and negative ranks**	105.0, 0.000
**Sum of signed ranks (W)**	105
**Number of pairs**	14
**Number of ties (ignored)**	0
**Median of differences**	
**Median**	1261

***: statistically significant.

## Data Availability

Data are available upon reasonable request to the corresponding author.
